# Diazotroph Diversity in the Sea Ice, Melt Ponds, and Surface Waters of the Eurasian Basin of the Central Arctic Ocean

**DOI:** 10.3389/fmicb.2016.01884

**Published:** 2016-11-23

**Authors:** Mar Fernández-Méndez, Kendra A. Turk-Kubo, Pier L. Buttigieg, Josephine Z. Rapp, Thomas Krumpen, Jonathan P. Zehr, Antje Boetius

**Affiliations:** ^1^HGF-MPG Group for Deep Sea Ecology and Technology, Alfred Wegener Institute Helmholtz Centre for Polar and Marine ResearchBremerhaven, Germany; ^2^HGF-MPG Group for Deep Sea Ecology and Technology, Max Planck Institute for Marine MicrobiologyBremen, Germany; ^3^Department of Ocean Sciences, University of California at Santa Cruz, Santa CruzCA, USA; ^4^Sea Ice Physics Section, Climate Sciences Department, Alfred Wegener Institute Helmholtz Centre for Polar and Marine ResearchBremerhaven, Germany

**Keywords:** nitrogen fixation, *nifH*, Arctic, non-cyanobacterial diazotrophs, sea ice, bacterial diversity

## Abstract

The Eurasian basin of the Central Arctic Ocean is nitrogen limited, but little is known about the presence and role of nitrogen-fixing bacteria. Recent studies have indicated the occurrence of diazotrophs in Arctic coastal waters potentially of riverine origin. Here, we investigated the presence of diazotrophs in ice and surface waters of the Central Arctic Ocean in the summer of 2012. We identified diverse communities of putative diazotrophs through targeted analysis of the *nifH* gene, which encodes the iron protein of the nitrogenase enzyme. We amplified 529 *nifH* sequences from 26 samples of Arctic melt ponds, sea ice and surface waters. These sequences resolved into 43 clusters at 92% amino acid sequence identity, most of which were non-cyanobacterial phylotypes from sea ice and water samples. One cyanobacterial phylotype related to *Nodularia* sp. was retrieved from sea ice, suggesting that this important functional group is rare in the Central Arctic Ocean. The diazotrophic community in sea-ice environments appear distinct from other cold-adapted diazotrophic communities, such as those present in the coastal Canadian Arctic, the Arctic tundra and glacial Antarctic lakes. Molecular fingerprinting of *nifH* and the intergenic spacer region of the rRNA operon revealed differences between the communities from river-influenced Laptev Sea waters and those from ice-related environments pointing toward a marine origin for sea-ice diazotrophs. Our results provide the first record of diazotrophs in the Central Arctic and suggest that microbial nitrogen fixation may occur north of 77°N. To assess the significance of nitrogen fixation for the nitrogen budget of the Arctic Ocean and to identify the active nitrogen fixers, further biogeochemical and molecular biological studies are needed.

## Introduction

Arctic marine ecosystems are rapidly changing due to climate change (Wassmann et al., 2011). In summer 2012, Arctic sea ice extent declined to its minimum ever recorded (Parkinson and Comiso, 2013), following the general trend in Arctic sea-ice cover decline (Stroeve et al., 2012). This led to vast areas of water being exposed to higher light intensities, which enhances phytoplankton primary production (Arrigo and van Dijken, 2015). Primary production in this highly stratified ocean is generally limited by nitrogen, most of which is delivered in the form of nitrate via the inflow of Pacific and Atlantic waters (Codispoti et al., 2013). Riverine input of nitrogen, especially from the Lena river that discharges into the Laptev Sea, may be another important source of nitrate in the Eurasian Basin (Holmes et al., 2011), alongside atmospheric deposition by snowfall (Beine et al., 2003), recycling of organic matter, and nitrogen fixation in ice masses. These different nitrogen sources should be balanced by nitrogen sinks, such as primary production and export, as well as microbial denitrification along the broad continental shelves that surround the Arctic Ocean (Devol et al., 1997; Rysgaard et al., 2004). However, current nutrient budgets indicate an imbalance of Arctic nitrogen supply, demand and export, suggesting that nitrogen fixation may have a key role in closing the budget (Torres-Valdés et al., 2013).

The biological fixation of gaseous dinitrogen (N_2_) by diazotrophs is an important source of bioavailable nitrogen in nutrient-limited pelagic ecosystems (LaRoche and Breitbarth, 2005), thereby influencing primary productivity and carbon export to the seafloor (Codispoti et al., 2001; Arrigo, 2005). Nitrogen fixation rates in surface waters of tropical, subtropical and some temperate oceans are primarily driven by diazotrophic cyanobacteria (Langlois et al., 2008; Moisander et al., 2010; Turk-Kubo et al., 2012). Non-cyanobacterial diazotrophs have also been detected in numerous marine, pelagic environments (Falcón et al., 2004; Riemann et al., 2010; Farnelid et al., 2011; Moisander et al., 2014), although their contribution to oceanic nitrogen fixation remains poorly understood (Turk-Kubo et al., 2014). In contrast to temperate and tropical seas, the specific contribution of nitrogen fixation and its key microbial agents in ice-covered seas is not well known (Tremblay et al., 2009; Blais et al., 2012; Luo et al., 2012; Torres-Valdés et al., 2013). This study aims at providing a first survey of the nitrogen-fixing potential of the eastern Central Arctic Ocean.

Several factors are thought to constrain oceanic nitrogen fixation: low temperatures (Brauer et al., 2013), high dissolved oxygen, high N:P ratios, and iron or phosphate limitation (Paerl and Zehr, 2000; Mills and Arrigo, 2010; Riemann et al., 2010; Monteiro et al., 2011). The generally low temperatures and the high dissolved oxygen in Arctic waters (Bates et al., 2014) support the long-held assumption that nitrogen fixation is unlikely in the Arctic Ocean. However, diazotrophs have developed strategies to withstand freezing and high salinity by producing antifreeze proteins (Schmidt et al., 1991), and to avoid oxygen by developing endo-symbiosis, like *Rhizobiales*, which include mainly *Alpha-proteobacteria* and *Beta-proteobacteria* and have been identified also in polar soils and frost flowers (Bordeleau and Prévost, 1994; Bowman et al., 2013). Symbiotic associations between diatoms and diazotrophs have been described in other oceans (Villareal, 1992; Foster et al., 2011). We hypothesize that sea-ice related environment of the Eurasian basin of the Central Arctic Ocean might hold diazotrophs.

Arctic waters often have low N:P ratios (Tremblay et al., 2008), which have been hypothesized to favor diazotrophs (Tyrrell, 1999). They are relatively rich in iron (1–3 nM) from river input (Klunder et al., 2012), which could well support the iron demand of nitrogen fixing enzymes. Nitrogen fixation rates and putative diazotrophs from riverine origin (microorganisms containing at least the *nifH* gene) have been identified close to the Mackenzie River and in the water column of the Canadian Arctic shelves (Farnelid et al., 2011; Blais et al., 2012). Relatively high cyanobacterial *nifH* gene diversity (e.g., *Trichodesmium* and *Cyanothece*) has also been described in the sea ice and waters of the Fram Strait in the Eurasian Basin (Díez et al., 2012). In contrast, to date there is no record of marine diazotroph occurrence in the Central Arctic Ocean north of 76°N. Non-diazotrophic cyanobacteria, such as *Prochlorococcus*, which are very abundant in other oceanic regions (Partensky and Hess, 1999), are rare in polar marine waters (Vincent, 2000; Lovejoy and Potvin, 2010) although they appear in both polar regions (Nadeau et al., 2001). Diazotrophic cyanobacteria, which represent a small percentage of marine cyanobacteria, are also the most common autotrophic diazotroph in other oceans (Moisander et al., 2010; Luo et al., 2012) and on glaciers (Yallop et al., 2012), but their diversity and abundance in the marine environments of the polar regions is still understudied.

In the Arctic Ocean, only a few *nifH* gene surveys have been conducted in the past decade, and they focused on coastal regions. In this study, we investigated *nifH* gene diversity in different Arctic environments, including Arctic sea ice and surface water from under-ice and open water locations. To assess diazotroph diversity and discover specific phylotypes that might be unique for the Arctic marine environment, we chose a targeted analysis of the *nifH* gene, coding for the iron protein of the nitrogenase enzyme. In addition, we used molecular fingerprinting analyses of the intergenic spacer region of the rRNA operon to characterize the complete bacterial community, and to reveal the dissimilarity patterns between different sea-ice related environments. Furthermore, we measured the physical-chemical properties of each environment type and we included data about the origin of the sea ice, measured by its drift trajectory.

These datasets allowed us to assess the distribution and diversity of diazotrophs in melt pond, sea-ice and surface waters of the Central Arctic and to evaluate the genetic nitrogen-fixing potential of the microbial communities. Specifically, we addressed the hypotheses that (1) putative diazotrophs of the Central Arctic occupy a niche in the nitrogen-limited sea ice and waters of the Eurasian Basin, (2) they originate from coastal areas such as the Laptev Sea where the ice is formed and transported with the Transpolar Drift, and (3) the Central Arctic marine sea-ice related diazotrophs are distinct from other cold-adapted diazotrophic communities and those of adjacent oceanic regions.

## Materials and Methods

### Sampling

**FIGURE 1:**
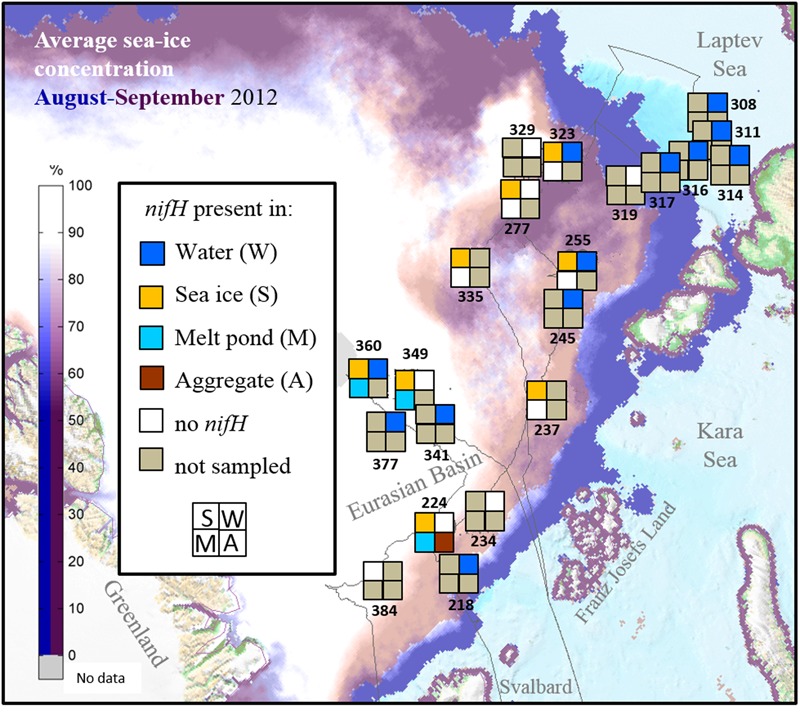
**Stations sampled for *nifH* analysis in the Eurasian Basin of the Central Arctic during summer 2012.** Station numbers correspond to those in Supplementary Table S1. Each square correspond to one environment. If a particular environment was not samples at one station it appears in gray. If no *nifH* gene could be amplified it is colored in white, and if *niH* genes could be amplified in the color corresponding to each environment (Dark blue = water, Yellow = sea ice, Light Blue = melt pond and Brown = algal aggregates). The thin gray line corresponds to the cruise track. Average sea-ice concentration data source: www.meereisportal.de.

A total of 53 samples were taken for this investigation during the ARKXXVII/3 cruise to the Central Arctic (77–88°N and 30–133°E) from the 7th of August to the 30th of September, 2012, onboard the German icebreaker R/V POLARSTERN (Supplementary Table S1). Sea ice, melt ponds and seawater were sampled at 9 ice stations and 13 water stations located in different parts of the central Eurasian Basin. Surface water samples from ice-covered waters (*n* = 13) were differentiated from those from open water from the Laptev Sea region (*n* = 6) for the analysis. Our samples come from a wide range of ice conditions and nutrient regimes (**Figure 1**). Samples of rapidly melting sea ice (*n* = 21) were taken using an ice corer (Kovacs Entreprise, Roseburg, OR, USA) and cut into two sections (top and bottom) prior to being transferred to plastic containers that had been rinsed with ultrapure water and ethanol and melted in the dark at 4°C. Samples of melt pond water (*n* = 8) and algal aggregates (*n* = 5) found in melt ponds were collected using a hand pump (Model 6132-0010, Nalgene, Penfield, NY, USA) and stored in clean Nalgene bottles. Samples of water under the ice (*n* = 5) were collected using a peristaltic pump (Masterflex^®^ E/S^TM^ portable sampler, 115 VAC, Oldham, UK). Surface water (2–5 m depth) samples at all other stations (*n* = 13) were collected with a rosette sampler equipped with Niskin bottles and a Conductivity Temperature Depth (CTD) profiler (Sea-Bird Electronics Inc., Bellevue, WA, USA). In addition, ultrapure water was sampled to check for possible contaminations from the onboard water purification system (Milli-Q Gradient A10, Millipore) when using this water to rinse the sampling equipment. Between 0.3 and 2 L volume of each sample were filtered through Sterivex filters of 0.2 μm pore size (Durapore, Milipore, Darmstadt, Germany) using a multichannel peristaltic pump (Model PD 51; Heidolph, Schwabach, Germany). Filters were stored at -80°C until further processing.

### Characterization of Central Arctic Environments

Temperature and salinity were measured in sea ice and melt pond water with a hand-held conductivity meter (315i with TetraCon electrode cell, WTW GmbH, Weilheim in Oberbayern, Germany), and in the water column with a CTD profiler. The data is available in the databases of the Data Publisher for Earth and Environmental Science, PANGAEA, doi: 10.1594/PANGAEA.819452 (Rabe et al., 2012). Concentrations of nitrate, phosphate and silicate were measured with a standard photometric method using a Technicon TRAACS 800 continuous flow auto analyzer (Technicon Corporation) as described in Fernández-Méndez et al. (2015). Raw nutrient concentrations and integrated stocks are available in PANGAEA doi: 10.1594/PANGAEA.834081 (Fernández-Méndez et al., 2014a; Bakker, 2014, unpublished).

Data concerning ice thickness and melt pond coverage are stored in PANGAEA, doi: 10.1594/PANGAEA.803221 (Hendricks et al., 2012). The origin of sea ice was determined using ice drift information obtained from satellites. In this study, two different sets of ice drift products were used: The first data set, Polar Pathfinder Sea Ice Motion Vectors (Version 2) obtained from the National Snow and Ice Data Center (NSIDC) was chosen because of its year round availability. We used it to calculate ice drift trajectories during summer months (June–August). The second data set, sea ice motion provided by the Center for Satellite Exploitation and Research (CERSAT) at the Institut Francais de Recherche pour l’Exploitation de la Mer (IFREMER), shows a good performance on the Siberian shelf (Krumpen et al., 2013) and was therefore used to complement the calculation of ice drift trajectories between September and May. To determine drift trajectories and source areas of sampled sea ice a specific ice area is tracked backward until: (a) the ice reaches a position next to a coastline, (b) the ice concentration at a specific location reaches a threshold value of (>15%) when ice parcels are considered lost, or (c) the tracking time exceeds 4 years. A more detailed method description is provided in Krumpen et al. (2016).

### *nifH* Gene Molecular Analysis

Total community DNA was extracted using the DNeasy Plant Mini Kit (QIAGEN, Valencia, CA, USA) and the QIAcube extraction instrument following the manufacturer’s instructions (Bombar et al., 2013). To amplify the *nifH* gene, a nested polymerase chain reaction (PCR) employing a high number of amplification cycles (*n* = 50) with two sets of degenerate primers was used since it is a high-sensitivity method (Zehr and Turner, 2001). The four degenerate primers used, *nifH*1-*nifH*4, cover >94% of available *nifH* sequences, allowing our approach to access a high percentage of the known diazotroph diversity (Gaby and Buckley, 2012) at the limited availability of sample volume in this study. PCR amplifications were performed in a MyCycler Thermal Cycler (BioRad, Berkeley, CA, USA). The first PCR amplification of *nifH* in each sample was performed with 2 μl of DNA template in 24 μl of PCR reaction containing 4 mM MgCl_2_, 0.4 mM dNTPs, 10X Buffer, 1.25 U (0.2 μl) Platinum TaqDNA Polymerase (Invitrogen) and 0.5 μM of the forward *nifH*3 primer (5′-ATR TTR TTN GCN GCR TA-3′) and reverse *nifH*4 primer (5′-TTY TAY GGN AAR GGN GG-3′) (Zehr and Turner, 2001). Amplifications proceeded with 25 cycles of 3 min at 95°C, 30 s of denaturation at 95°C, 30 s of annealing at 55°C and 45 s of elongation at 72°C. The second amplification procedure was performed as described above, save the use of 1 μl of the PCR product from the first amplification process as the template and the forward *nifH*1 primer (5′-TGY GAY CCN AAR GCN GA-3′) and reverse *nifH* primer (5′-ADN GCC ATC ATY TCN CC-3′) (Zehr and Turner, 2001). In addition, the annealing temperature was increased to 57°C. In both steps, negative controls were performed using milliQ water in place of the DNA template.

A 15 μl aliquot of the PCR products from the second amplification was used for electrophoresis separation (1,4% low-melt agarose gel). The DNA band of the appropriate size (360 bp) was extracted with a gel extractor (X-tracta gel extractor, USA Scientific, Ocala, FL, USA). The gel was purified with a QIAquick Gel Extraction Kit (Qiagen) and the DNA cloned with a TOPO^®^ TA Cloning^®^ Kit for Sequencing with One Shot^®^ TOP10 Chemically Competent *E. coli* (Invitrogen) following the manufacturer’s guidelines. Dependent on outcomes of the cloning process, plasmids from between 12 and 48 clones per sample were purified using the Millipore Montage Plasmid Miniprep_96_KitsMiniprep kit (Millipore, Darmstadt, Germany) and the inserts were sequenced using the Sanger method (Sanger et al., 1977) at the University of California, Berkeley.

Nucleic acid sequence data were trimmed and quality checked using the Sequencher^®^ sequence analysis software (Gene Codes Corporation, Ann Arbor, MI, USA). Quality-controlled sequences were imported into the software program ARB (Ludwig et al., 2004), translated to amino acid sequences and imported into a publically available, curated GenBank database containing all *nifH* sequences submitted to the nr database that have been validated to be *nifH* based on analysis (Heller et al., 2014; April 2014 release). Amino acid sequences were aligned using the program HMMER which contains a Hidden Markov Model from the protein family database PFAM (Finn et al., 2010). Subsequently, the nucleotide sequences were re-aligned according to the aligned amino acid sequences using ARB. To confirm that the sequences were *nifH* gene, the alignments were visually checked for conserved regions. Our 572 confirmed *nifH* sequences included one sequence from the PCR blank and 28 sequences from the ultrapure water used to rinse the plastic containers where the samples were stored. These sequences clustered at >94% amino acid sequence identity with 15 sequences from our environmental samples, so these were considered putative contaminants and removed from further analyses. We also checked if any of the retrieved sequences were closely related to other sequences reported as contaminants on other studies. The 45 contaminant amino acid sequences reported in the April 2014 *nifH* database (Farnelid et al., 2009, 2013) were all less than 90% similar to our sequences retrieved from the environment. Despite these checks, one cannot exclude the possibility that some of the sequences reported might be contaminants. From the 529 confirmed environmental *nifH* sequences, a total of 43 clusters with ≥92% amino acid sequence identity were identified using the CD-HIT program suite (Huang et al., 2010). Each cluster was represented by one representative sequence. A maximum likelihood tree of partial *nifH* sequences was built in RAxML version 8.1 (Stamatakis, 2014). This tree included the 43 representative sequences (92% amino acid similarity using the matrix BLOSUM62) of our *nifH* Arctic sequences and their closest cultivated relatives. We used the RAxML function *auto prot* to determine the most appropriate protein substitution model. Following the function’s recommendation, we used the LG matrix substitution model to determine relatedness (represented by branch length) and a custom mask for the *nifH* amplicon region (Mask Makyyy in the ARB publicly available database). The stability of the RAxML phylogenetic reconstruction was assessed by bootstrapping 1000 times with RAxML’s *-b* function. The tree and its associated metadata were visualized using ITOL (Letunik and Bork, 2007). The phylogenetic affiliation of each *nifH* cluster, defined by CD-HIT, was determined based on the phylogenetic affiliation of the closest cultivated relative, which have been assigned according to the convention suggested by Zehr et al. (2003) and provided in the curated *nifH* database (Heller et al., 2014).

For the comparison of different regions, sequences from nine independent studies, including the present one, were selected from studies submitted to the GenBank database prior to April 2014. All available Arctic sequences from different environments were selected, as well as all Antarctic sequences, which were exclusively from lake microbial mats. In addition, sequences from one study in the North Atlantic (constrained to surface water samples as in our study) and one in the subtropical Atlantic (water samples collected at 8 m depth) were selected for further comparison outside of the polar regions. In total, 1523 sequences were chosen to characterize the diazotroph community structure from: the Central Arctic (this study) (*n* = 572), the coastal Canadian Arctic (Blais et al., 2012) (*n* = 21), the coastal Eurasian Basin (Díez et al., 2012) (*n* = 69), the Arctic tundra soil (Izquierdo and Nüsslein, 2006) (*n* = 24), the Antarctic ice shelf microbial mats in lakes (Olson et al., 1998; Jungblut and Neilan, 2010) (*n* = 15+43), the subtropical Atlantic Ocean (Langlois et al., 2005) (*n* = 175), and the North Atlantic Ocean (Turk et al., 2011) (*n* = 604). All studies except Díez et al., 2012 used the same primers as used in this study. Nucleotide sequences from these studies were clustered using CD-HIT EST (Li and Godzik, 2006) at 97% nucleotide similarity (Turk-Kubo et al., 2014). From the 243 representative sequences (of 1523 initial sequences), 86 originated from *nifH* genes present in the Central Arctic, 19 from the Canadian Arctic, 20 from the Eurasian Basin, 16 from the Arctic tundra, 25 from the Antarctic microbial mats, 51 from the North Atlantic, and 26 from the subtropical Atlantic. The representative sequences at 97% nt identity retrieved from the Central Arctic sea-ice related environments as well as the ultrapure water blanks were submitted to GenBank and assigned accession numbers KT354077-KT354180. Only five of the CD-HIT clusters represented sequences from both the North Atlantic and the subtropical Atlantic and one CD HIT cluster represented sequences from the Eurasian Basin and the Subtropical Atlantic. All other CD-HIT clusters represented sequences exclusively from one oceanic region. A maximum likelihood tree of these 243 representative sequences was built using RAxML v8.1. The best performing model was the WAG matrix substitution model. The stability of the RAxML tree was assessed by bootstrapping 1000 times with RAxML’s *-b* function. The tree and its associated metadata were visualized using ITOL (Letunik and Bork, 2007).

### Intergenic Spacer 16SrRNA Molecular Analysis

The bacterial community structure was investigated targeting the Intergenic Spacer (ITS) region of the 16SrRNA by Automated Ribosomal Intergenic Spacer Analysis (ARISA) (Fisher and Triplett, 1999) in the 26 samples where *nifH* could be amplified. ARISA was chosen as a rapid and effective method to analyze bacterial community structure across this large dataset. Results of this method were previously shown to be coherent with diversity patterns retrieved using next generation sequencing approaches (e.g., Gobet et al., 2014). Each ARISA PCR contained 2.5 μl of 10x reaction buffer S (PEQLAB Biotechnologie GmbH, Erlangen, Germany), 1 μl of 25 mM MgCl_2_ (PEQLAB Biotechnologie GmbH, Erlangen, Germany), 0.625 μl of a 10 mM dNTP mix (PEQLAB Biotechnologie GmbH, Erlangen, Germany), 0.75 μl of 3 mg ml^-1^ bovine serum albumin (Sigma-Aldrich Chemie Gmbh; Munich, Germany), 0.25 μl of 40 μM universal forward primer ITSF (5′-GTCGTAACAAGGTAGCCGTA-3′) (Biomers.net, Ulm, Germany), labeled with 6-carboxyfluorescein (FAM), 0.25 μl of 40 μM ITSReub reverse primer (5′-GCCAAGGCATCCACC-3′) (Biomers.net, Ulm, Germany), 0.25 μl of 5 units μl^-1^ Taq polymerase (PEQLAB Biotechnologie GmbH, Erlangen, Germany) and approximately 10 ng environmental DNA as determined by spectrophotometry (Infinite^®^ M200 NanoQuant, Tecan Group Ltd., Switzerland). PCR water was added to each reaction mix to a final volume of 25 μl. All reactions were conducted in triplicate. PCR conditions were set to 3 min at 94°C, 30 cycles of first 94°C for 45 s, 55°C for 45 s, and 72°C for 90 s followed. Final extension time was 5 min at 72°C.

PCR performance was examined using gel electrophoresis and the length of successfully amplified and purified ITS fragments was analyzed via capillary electrophoresis on an ABI Prism 3130 XL – Genetic Analyzer (Applied Biosystems, Carlsbad, CA, USA). Signals were evaluated using the GeneMapper Software v3.7 (Applied Biosystems, Carlsbad, CA, USA) and subsequent generation of operational taxonomic units (OTUs) was done with custom R scripts^1^ as previously described (Ramette, 2009).

### Statistical Analysis and Ordination

All statistical tests were performed in R version 3.1.1 (R Core Team, 2015). Differences in diazotroph community structure – based on the presence or absence of *nifH*-derived operational taxonomic units – were expressed using the Jaccard dissimilarity measure, which was computed as 2*B*/(1+*B*), where *B* is the Bray–Curtis dissimilarity measure:

$$B=\frac{\sum_{i-1}^{S}\left(x_{\text{ij}}-x_{\text{ik}}\right)}{\sum_{i-1}^{S}\left(x_{\text{ij}}+x_{\text{ik}}\right)}$$

and visualized by non-metric multidimensional scaling (NMDS) using the metaMDS routine of the “vegan 2.3” package (Oksanen et al., 2013). Differences in the total bacterial community structure – represented through variations in the internal transcribed spacer region (ITS) fingerprint – were similarly visualized, but calculated using the Bray–Curtis dissimilarity measure (Legendre and Legendre, 1998). Guided by the Shepherd stress of our NMDS results, we chose to ordinate ranked dissimilarities from both analyses in two-dimensional space. In each plot, dissimilarity between samples is approximated by the distances between the points representing them. Stress values reflect the degree of correspondence between the distances between points in the NMDS plot and values in the original dissimilarity matrix. An analysis of similarity (ANOSIM) was used to evaluate how strongly our representations of bacterial community structure differed between *a priori* defined groups. The groups defined were: Laptev Sea (*n* = 4), Surface Waters (*n* = 6), Melt Ponds (*n* = 3), Ice Bottom (*n* = 3), Ice Top (*n* = 8), Brown ice (*n* = 1), and Aggregate (*n* = 1). Groups with one sample only were excluded from the analysis. First, we performed an omnibus test followed by a pairwise ANOSIM as *post hoc* testing. Individual *p*-values were Bonferroni-corrected. A Mantel test was performed using the mantel routine of the “vegan 2.3” package to compare the two dissimilarity matrices (*nifH* and ARISA).

To complement our dissimilarity-based methods, which may confound location and spread (Warton et al., 2012), redundancy analysis (RDA) using type I scaling was used to estimate the degree of linear association between variation in the diazotroph community and temperature, salinity, nitrate, phosphate and silicate. These environmental variables were standardized by *z*-scoring prior to use in RDA. The response data was Hellinger transformed to standardize it (Legendre and Gallagher, 2001), using the *decostand* function of the “vegan 2.3” package. The significance of the RDA analysis was calculated using ANOVA and the residuals were plotted in a histogram (Supplementary Figure S3).

To compare the diazotroph community of the Central Arctic Ocean described in this study to the diazotroph community from Arctic adjacent oceanic regions (Canadian and Eurasian Arctic shelves), other polar environments (Antarctic lake microbial mats and Arctic tundra soil), and other oceanic regions (North and Subtropical Atlantic), we performed phylogenetic distance analysis (Unifrac) (Lozupone et al., 2006) on the maximum likelihood phylogenetic tree containing only representative sequences from those environments (CD-HIT clustering at a 97% nucleotide similarity threshold). To calculate dissimilarities based on phylogenetic distances between the diazotrophic communities we used the package “phyloseq” in R (McMurdie and Holmes, 2013). Principal coordinate analysis (PCoA) was used to ordinate Unifrac distances using the functions *cmdscale()* and *ordiplot()* in the R package “vegan 2.3.” An automatic correction for negative eigenvalues was used. Rarefaction curves were computed across Hill numbers (Chao et al., 2014) using the iNEXT package (Hsieh et al., 2016), both for 92% amino acid similarity representative sequences in the different sea-ice related environments of the Central Arctic, as well as for the 97% nucleotide identity representative sequences in the different oceanic regions. Hill number 0 refers to species richness, 1 to the number of ’typical’ species in the community (exponential of Shannon entropy), and 2 to the number of very abundant species in a community (inverse Simpson).

## Results

### Characterization of Arctic Diazotroph Communities Based on *nifH* sequences

**FIGURE 2:**
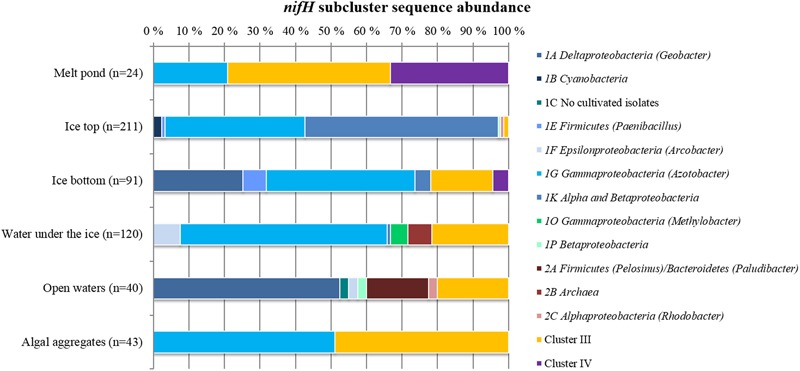
**Relative abundances of different *nifH* subclusters in Central Arctic environments.** The number of sequences retrieved from each environment is shown in parenthesis. Open waters correspond to the region close to the Laptev Sea.

**FIGURE 3:**
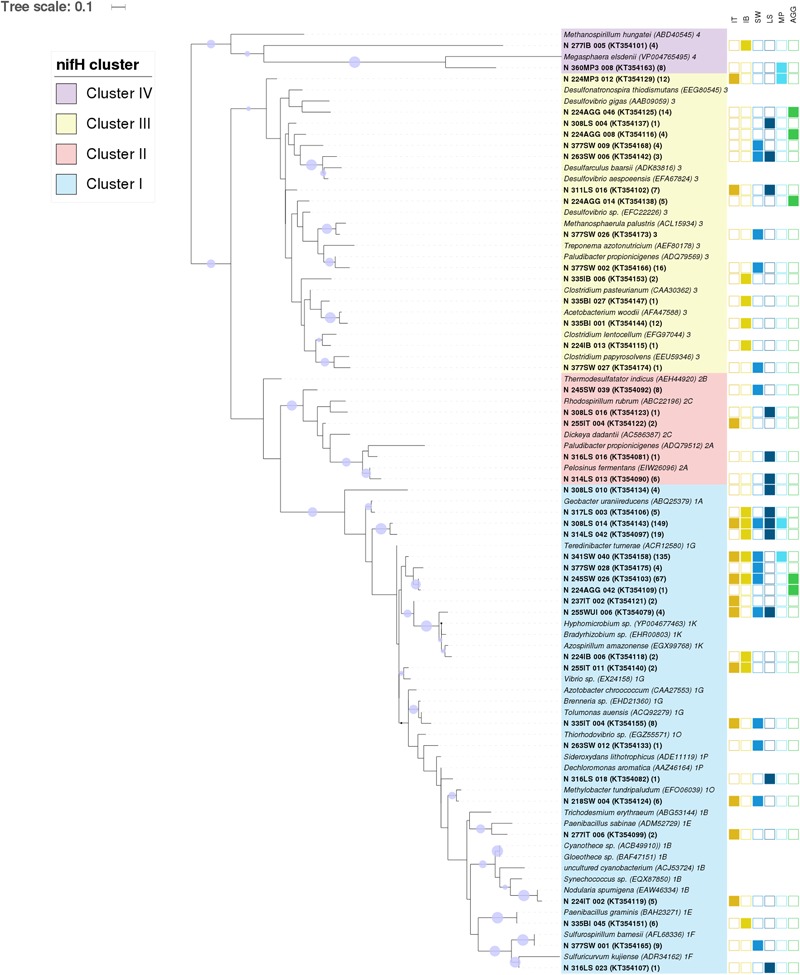
**Central Arctic *nifH* gene diversity in summer 2012.** Maximum likelihood phylogenetic tree of *nifH* partial amino acid sequences (104 positions) containing representative sequences from the Central Arctic (bold) and their closest cultivated relatives (italics). The Central Arctic *nifH* amino acid sequences were clustered using CD-HIT at 92% amino acid similarity to determine representative sequences. The origin of our Central Arctic representative sequences is indicated in the name. The number of sequences represented by each CD-HIT subcluster are indicated in brackets. The station number is followed by the environment from which it originates: Ice Top (IT), Ice Bottom (IB), Brown Ice (BI), Melt Pond (MP), Algal aggregate (AGG), Water under the ice (WUI), Surface water (SW), and Laptev Sea (LS). The *nifH* subcluster assignment for each cultivated diazotroph is indicated after the name. Branch lengths were determined using the LG protein substitution model as recommended by RAxML Version 8. The percentage of calculated trees in which sequences clustered together in the bootstrap test (1000 replicates) is shown next to the branch as a purple circle when greater than 50% (smallest circle corresponds to 50% and greater to 100%). GenBank accession numbers for the 43 representative sequences are in parenthesis after the sequence name.

During our study in summer 2012, *nifH* gene fragments were amplified from 26 of the 53 samples collected in different Arctic environments: sea ice, melt ponds and surface water column (**Figure 1**). A total of 529 sequences were retrieved, 40% of them from the upper part of the sea ice and only 5% from melt ponds (**Table 1**; Supplementary Table S1). These sequences were clustered into 43 clusters at 92% amino acid similarity and were distributed across all four main *nifH* clusters I–IV as defined by Zehr et al. (2003), including both cyanobacterial and non-cyanobacterial phylotypes (**Figures 2** and **3**). Central Arctic sequences affiliated with Cluster I contained mainly *Proteobacteria* (1G and 1K), *Firmicutes* (1E), *Cyanobacteria* (1B) and several uncultivated microorganisms. Cluster II contained *Proteobacteria* (2C), *Firmicutes* (2A), and members of the Archaea (2B). Cluster III contained putative anaerobes including sulfate reducing genera of the *Deltaproteobacteria*, and genera such as *Clostridium*. Cluster IV contained *nifH* paralogs that are thought to function in metabolic processes other than nitrogen fixation (e.g., Young, 2005; Staples et al., 2007).

**Table 1:** Number of samples screened for *nifH* gene amplification and confirmed *nifH* gene sequences retrieved.

Environment	Total Samples	Samples with *nifH*	Sequences retrieved
Melt ponds	8	3	24
Ice top	8	8	211
Ice bottom	13	4	91
Water under the ice	13	6	120
Surface open waters Laptev Sea	6	4	40
Algal aggregates	5	1	43
Total	53	26	529

The vast majority of sequences retrieved across the different environments of the Central Arctic Ocean belonged to non-cyanobacterial diazotrophs of Cluster I (**Figure 2**). In this cluster, 53% of the sequences belonged to the subcluster 1G that contains sequences from genera such as *Azotobacter, Brenneria, Teredinibacter*, and *Pseudomonas.* Subcluster 1K comprised 29% of the sequences, containing both *Alpha-* and *Beta-proteobacteria* such as *Bradyrhizobium* and *Azospirillum* sp. From subcluster 1B, which contains exclusively cyanobacterial diazotrophs, only five sequences were amplified from the upper layer of the sea ice (top 50 cm of a snow-free core) at ice station 224 (Subcluster 1B, Representative sequence N_224IT_002 in **Figure 3**). These sequences were all closely related (≥92% amino acid sequence similarity) to *Nodularia*. Cluster III sequences accounted for up to 20% of the sequences retrieved by clone libraries from all samples together (**Figure 2**). Only 12 sequences corresponding to *nifH* paralogs (Cluster IV) were retrieved from sea-ice and melt pond samples, but they were not closely related to any cultivated organism (**Figure 3**). Since they do not provide any insight into nitrogen fixation capabilities of the Arctic microbial community, we will not develop them further.

### Presence of *nifH* Genes in the Central Arctic and Link to Temperature and Nutrients

Two of the main environmental factors that might affect the presence of diazotrophs, temperature and nutrient concentrations, were quite variable in the environments and stations sampled. Seawater temperatures ranged from -1.7°C below the ice and up to 3°C in open waters of the Laptev Sea shelf (Supplementary Figure S1). Sea-ice temperatures were between -0.2 and -1.8°C and melt pond temperatures between -0.7 and 0.3°C. Nitrogen fixation genes from putative diazotrophs were amplified from samples along the entire temperature range sampled (**Figure 1**).

Regarding nutrients, nitrate concentrations in summer 2012 were very similar in sea ice and melt ponds ranging between 0.2 and 1.5 μM, while being more variable in surface waters ranging between 0.02 and 6.4 μM (with highest concentrations at stations 218 and 245). Phosphate, the most relevant nutrient for diazotrophs, was more variable in sea ice (0.02–1.95 μM) reaching its highest concentrations at ice station 224. Melt ponds had in general very low phosphate concentrations (0.04–0.2 μM) while in surface waters the concentrations were higher (0.15–0.49 μM). As indicated by the N:P molar ratio, during summer in the Central Arctic, two nutrient regimes were identified in the euphotic zone of the water column (Supplementary Figure S2A). All N:P molar ratios in surface waters were below Redfield (<16:1) indicating general nitrogen limitation. The Atlantic-influenced ice margin in the Nansen Basin and the Laptev Sea had N:P ratios around 10, while the more Central Arctic waters in the Amundsen Basin had N:P ratios below 5. In sea ice, nutrient concentrations were in general lower than in the water column and the integrated N:P ratios were more variable. N:P ratios at stations 335 and 349 were close to Redfield, while the rest ranged between 5 and 11 (Supplementary Figure S2B). In melt ponds the N:P ratio ranged between 1 and 16. The genes responsible for nitrogen fixation could be amplified in all environments regardless of their N:P ratio at the time of sampling.

**FIGURE 4:**
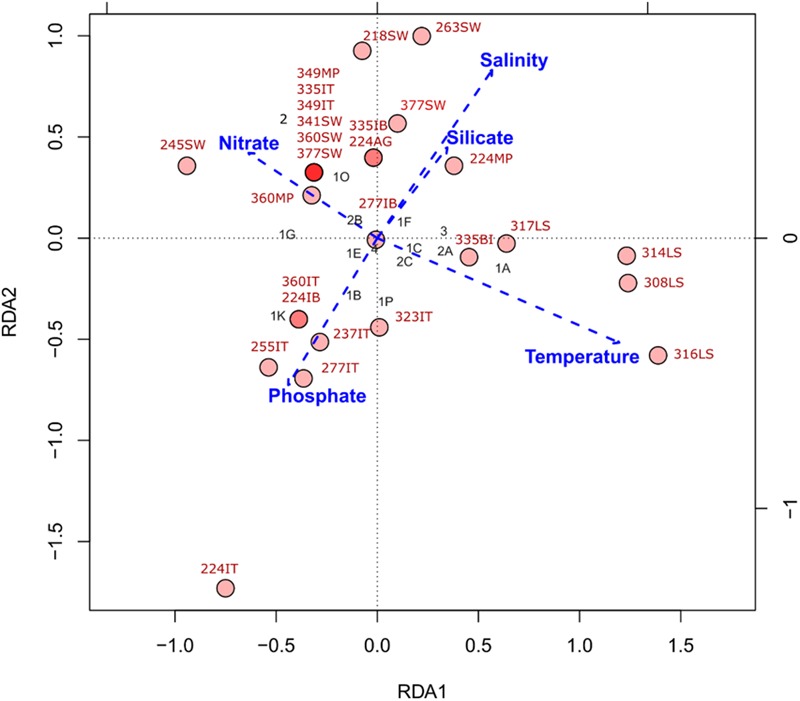
**Redundancy analysis (RDA) of *nifH* subcluster presence absence in Central Arctic samples and environmental variables.** Environmental variables: temperature, salinity, nitrate, phosphate and silicate were standardized by *z*-scoring prior to RDA. The red circles represent the different samples and the intensity of the color indicates if there is one (light red) or many (dark red) samples at that coordinate in the plot. Sample names in red correspond to those in Supplementary Table S1. The black labels correspond to the different subclusters of *nifH* (Cluster number 1, 2, 3, or 4; and the subcluster letter A-K). The explanatory variables constrained ∼57% of the variance in the *nifH* subclusters across samples. The significance of this type I scaling was 0.001 and the residuals were mostly distributed around zero (Supplementary Figure S3).

Our RDA suggests that our current set of explanatory variables are only able to account for just over half (∼57%) of the variation in our *nifH* subcluster data (**Figure 4**). Salinity and silicate increasing concentrations showed strong positive covariation, while phosphate concentrations showed a negative covariation with the other variables (**Figure 4**). Nitrate concentrations and temperature showed strong negative covariation. In general, *nifH* genes are found across a wide range of temperature and nutrient values, but with changing community structure. For example, Laptev Sea samples, containing sequences from subclusters 1A (*Deltaproteobacteria*) and 2A (*Firmicutes and Bacteroidetes*), were more associated with increased temperatures and decreased nitrate concentrations relative to the other samples analyzed (**Figure 4**). The presence of cyanobacterial *nifH* genes at the upper layer of the ice at station 224 (labeled as 224IT in **Figure 4**), is associated with the high phosphate concentrations.

### Diazotroph and Total Microbial Community Diversity Patterns in Different Environments in the Central Arctic

**FIGURE 5:**
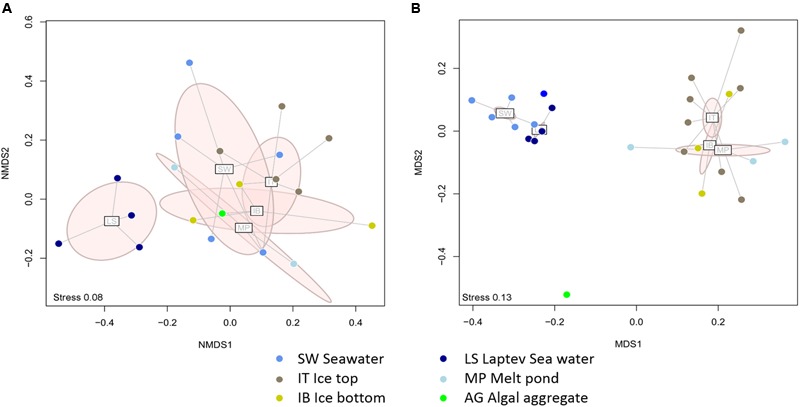
**Dissimilarities between the diazotrophic communities (A)** and the entire bacterial communities **(B)** of different Central Arctic environments. Laptev Sea (LS) samples are in dark blue, Ice Top (IT) in brown, Ice bottom (IB) in yellow, Surface waters (SW) in blue, Melt Ponds (MP) in light blue, and ice algal aggregate in green. Points within each group are connected to their group centroid with a spider diagram. The light pink ellipses show 95% dispersion of each group. **(A)** Two dimensional NMDS ordination plot of Jaccard dissimilarities based on the presence absence of *nifH* subclusters in each sample. The stress of the plot is 0.08. An ANOSIM *post hoc* test showed that the Laptev Sea samples are significantly different from the sea-ice environment samples (*R* = 0.46, *p* = 0.003). Individual p values were Bonferroni corrected. **(B)** Two dimensional NMDS ordination plot of Bray-Curtis dissimilarities based on the relative abundances of ARISA OTUs. The stress of the plot is 0.13. An ANOSIM *post hoc* test showed strong dissimilarities between the Surface Water and the Ice Top samples (*R* = 0.94, *p* = 0.005) as well as the Laptev Sea and Ice Top samples (*R* = 0.90, *p* = 0.014). Individual *p*-values were Bonferroni corrected.

Comparing the distribution of the *nifH* subclusters across the Central Arctic environments sampled, we observed differences between the Laptev Sea open waters and the sea-ice related environments. According to the dissimilarities between the *nifH* subclusters present in each sample, we detected a cluster of Laptev Sea samples that was distinctly separate from samples from sea ice environments (**Figure 5A**) (ANOSIM *R* = 0.46; *Post hoc* test *p* = 0.003). The rarefaction curve for each environment (Supplementary Figure S4A) shows that the Laptev Sea is under sampled, but the abundant types that we captured (represented in the panels for Hill numbers 1 and 2) were very different from the others. In addition, the 16S rRNA analysis also shows that the total bacterial community of the Laptev Sea is different from the sea-ice related environments (**Figure 5B**). However, in this case, the Laptev Sea clusters together with the other surface water samples, which is not the case for the diazotrophic community. The ice top environment and the Laptev Sea open water communities (ANOSIM *R* = 0.90; *p* = 0.014) showed moderate differences, as well as the ice top and the ice-covered surface waters (ANOSIM *R* = 0.94; *p* = 0.005).

Sea ice and Laptev Sea open waters had the largest number of unique representative sequences. These two environments had four *nifH* subclusters in common (1A, 1K, 1P, and 2C) (**Figure 2**). Most of the recovered *nifH* sequences in the top half of the ice belonged to subclusters 1G and 1K, which contain *Gamma-* and *Alphaproteobacteria*, respectively. We detected the greatest number of unique representative sequences in samples acquired from the lower portion of the ice, which contained sequences affiliated with subcluster 1A (*Deltaproteobacteria*) and Cluster III (**Figure 2**). Melt ponds, formed on top of the ice, only shared 1G subcluster sequences with the upper part of the ice, and additionally contained sequences from Cluster III (**Figure 2**). We detected sequences from subcluster 1G and from Cluster III – also found in our melt pond samples – in our samples of algal aggregates. These aggregates were primarily composed of algal species associated with sea ice. However, at a higher phylogenetic resolution, sequences in Cluster III differed between both environments (**Figure 3**).

More than half of the *nifH* sequences retrieved from water collected below the ice clustered together with sequences from the bottom part of the ice, an anticipated result because these two environments are in constant connection. However, the rest of the sequences detected in our water samples belonged to subclusters not detected in ice or melt pond samples: 1F (*Epsilonproteobacteria*), 1O (*Gammaproteobacteria*) and 2B (*Archaea*). The 1F subcluster also appeared in the open water samples from the Laptev Sea region (latitudes 77–79°N). The major subclusters present in our Laptev Sea open water samples were 1A and 2A. Subcluster 1A contains non-sulfate reducing *Deltaproteobacteria* such as *Geobacter* sp. and subcluster 2A contains fermenting bacteria from the genus *Pelosinus* and *Paludibacter*. *Deltaproteobacteria* from Cluster III composed around 20% of the sequences from open waters (**Figure 2**).

### Comparison of Central Arctic Diazotrophic Communities across Adjacent Polar Environments and Oceanic Regions

**FIGURE 6:**
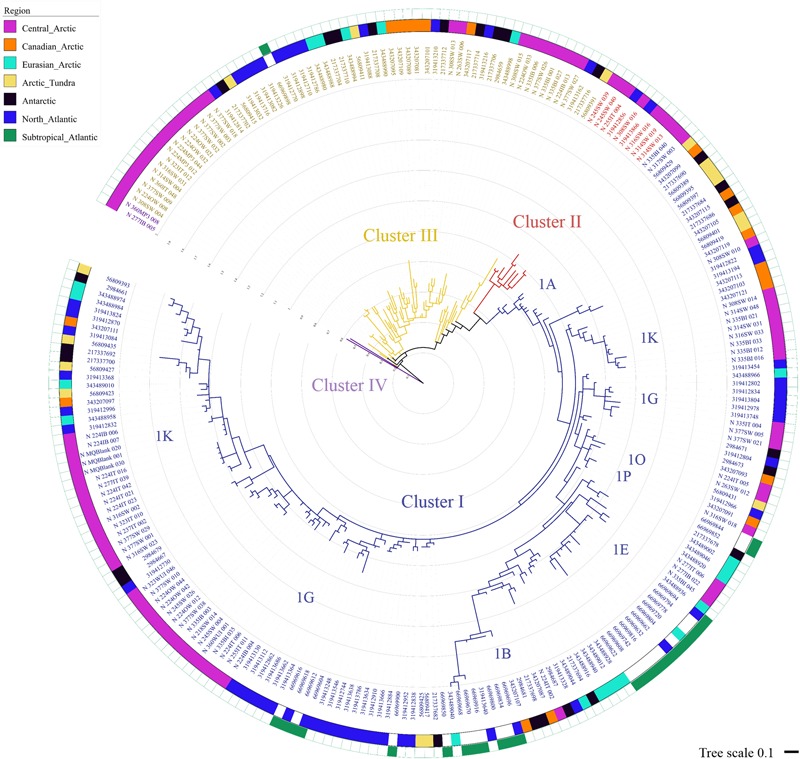
**Maximum likelihood phylogenetic tree of *nifH* partial amino acid sequences including sequences from the Central Arctic (this study), as well as other oceanic regions and similar cold environments.** Maximum likelihood tree of the 243 representative sequences clustered using CD-HIT EST (Li and Godzik, 2006) at 97% nucleotide similarity (Turk-Kubo et al., 2014) built using RAxML V8.1. The best performing model was the WAG matrix substitution model and we performed 1000 bootstraps. The tree and its associated metadata were visualized using iTOL (Letunik and Bork, 2007). The colored strips indicates the origin of the sequences represented by each representative sequence shown in the tree. From the 243 representative sequences, 86 contained sequences exclusively from the Central Arctic (pink), 19 from the Canadian Arctic (Blais et al., 2012) (orange), 20 from the coastal Eurasian Basin (Díez et al., 2012) (light blue), 16 from the Arctic tundra (Izquierdo and Nüsslein, 2006) (yellow), from the Antarctic ice shelf (Olson et al., 1998; Jungblut and Neilan, 2010) (black), 51 from the North Atlantic (Turk et al., 2011) (dark blue), and 26 from the subtropical Atlantic (Langlois et al., 2005) (green). Note that the North Atlantic and the Subtropical Atlantic shared five representative sequences and the Eurasian Basin and the Subtropical Atlantic share one. The four clusters of *nifH* classification are marked in blue (Cluster I), red (Cluster II), yellow (Cluster III) and purple (Cluster IV). Cluster I subclusters (1A, 1B, 1K, 1G, etc) are indicated close to their node.

**FIGURE 7:**
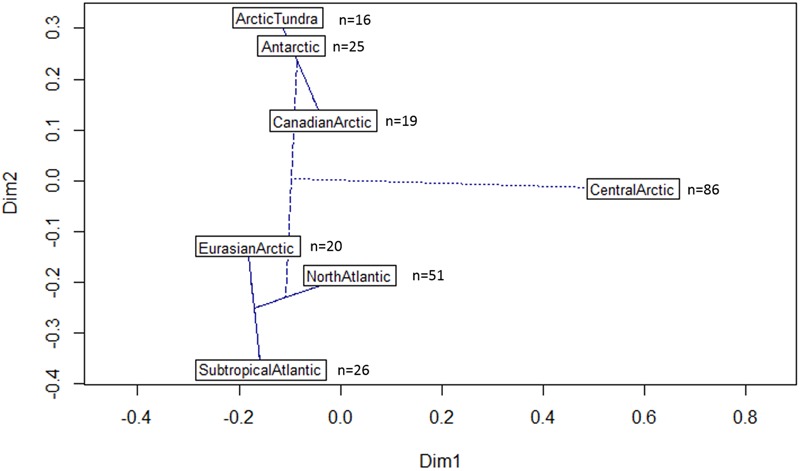
**Dissimilarities between different oceanic regions based on their *nifH* phylogenetic signature.** Plot of the first two principal coordinate axes of the Principal coordinates analysis (PCoA) derived from the Unifrac distances between different oceanic regions included in the maximum likelihood tree (**Figure 6**). The further away the samples are, the more dissimilar they are in terms of *nifH* phylogenetic signature. An automatic correction for negative eigenvalues was used. A cluster dendrogram is superimposed. The lines indicate the strength of the dissimilarity. Oceanic regions connected with a solid line have a dissimilarity less than 0.69 (Supplementary Table S2). The dashed line indicates a dissimilarity of 0.76 and the doted line between the Central Arctic and the subtropical Atlantic indicates the maximum dissimilarity of 0.85 (Supplementary Table S2). The number of representative sequences (97% nucleotide identity) from each oceanic region included in the analysis is indicated next to the region’s name.

The diazotrophic community of the Central Arctic is highly dissimilar to all other regions and environments we compared it to as unweighted unifrac distances were the highest (0.85–0.78), when the other regions were between (0.75–0.58) (Supplementary Table S2). At 97% nucleotide identity almost no sequences from different oceanic regions clustered together (**Figure 6**). Only five phylotypes were shared between the North Atlantic and the Subtropical Atlantic (**Figure 6**). The *nifH* diversity in sea ice, melt ponds and surface waters of the Central Arctic is different from the coastal Canadian Arctic marine diazotrophic community (Mackenzie river, Baffin bay) (**Figure 7**). Furthermore, it also differs from the sea ice, snow and water column communities from the coastal Eurasian Arctic (close to Svalbard) (**Figure 7**). The rarefaction curves show that the diversity in regions from which we acquired a greater number of sequences (i.e., the Central Arctic, the North Atlantic and the Subtropical Atlantic) appears to have been reasonably well sampled (Supplementary Figure S4B). However, the other oceanic regions are still in the rapid accumulation phase of their respective curves and deeper sequencing is likely to strongly affect their diversity profiles (Supplementary Figure S4B). We therefore place more interpretive weight on dissimilarities between our well-sampled regions (marked in the dissimilarities Supplementary Table S2), while noting that dissimilarities bearing on the Arctic tundra, Canadian Arctic, Eurasian Basin and Antarctic might be subject to change when the sampling effort is increased.

## Discussion

### Distribution of Diazotrophs in the Central Arctic Ocean

Confirming our first hypothesis, we found putative diazotrophs in the nitrogen-limited sea ice and waters of the Central Arctic Ocean (77–89°N). Most of the *nifH* sequences retrieved were non-cyanobacterial diazotrophs, mainly distributed between Cluster I and III as the bacterial phylotypes described in the marine Arctic studies by Farnelid et al. (2011), Blais et al. (2012), and Díez et al. (2012). All prominent nitrogen-fixing taxa at the subclade level present in other oceans were also present in the Central Arctic Ocean. However, most of the *nifH* genes amplified in the Central Arctic belonged to non-cyanobacterial diazotrophs, while cyanobacteria were under-represented. Previous Arctic diazotroph diversity studies also retrieved only a few cyanobacterial phylotypes. *Cyanothece* sp. and *Trichodesmium* sp., were found in the coastal Eurasian Basin in early May (Díez et al., 2012) and *Nostocales* sp., was found in the Beaufort Sea in July–August (Blais et al., 2012). The fact that we recovered only a single cyanobacterial phylotype in late summer, *Nodularia* sp., a cyanobacterial genus that contributes to nitrogen fixation in the Baltic Sea (Bostrom et al., 2007)., is consistent with the results of Farnelid et al. (2011), who observed that their Arctic station sampled in July (in Baffin Bay) only yielded non-cyanobacterial diazotrophs from Cluster III. This implies that cyanobacterial diazotrophs may not thrive in Central Arctic waters late in the productive season, far away from the coast line where there is riverine influence. However, *nifH* clone-library based approaches need to be interpreted with caution due to possible primer bias toward certain non-cyanobacterial diazotrophs (Turk et al., 2011; Turk-Kubo et al., 2015). Furthermore, the use of cyanobacteria-selective primers by Díez et al. (2012) might explain the high cyanobacterial diazotroph diversity reported in their study, compared to others.

The non-cyanobacterial diazotrophic community inhabiting the water column in the Central Arctic Ocean was dominated by Cluster I sequences: mainly *Alphaproteobacteria* (subcluster 1K) and *Deltaproteobacteria* (subcluster 1G) (**Figure 2**). While *Alpha-* and *Gammaproteobacteria* dominated non-cyanobacterial diazotrophic assemblages in the Atlantic and Pacific Oceans (Falcón et al., 2004; Langlois et al., 2005) and were present in lower abundances in the water column above the Canadian Arctic shelves (Blais et al., 2012). Sequences related to anaerobic bacteria (Cluster III) made up to 20% of under ice and open water communities in the Central Arctic (**Figure 2**). Cluster III sequences were once thought to be rare in brackish or marine surface waters (Moisander et al., 2007), but they are increasingly being recovered from ocean waters (Langlois et al., 2008; Turk-Kubo et al., 2014) including the Canadian Arctic shelf region (Farnelid et al., 2011).

Among the sea-ice related environments studied, floating aggregates are conglomerations of sea ice diatoms sticking together in a mucous matrix that can have an anoxic interior and, therefore, have the potential to provide an environment for anaerobic processes such as denitrification and nitrogen fixation (Fernández-Méndez et al., 2014b; Lehto et al., 2014). Therefore, it is important to study *nifH* diversity in this environment. It is difficult to infer phylogeny from Cluster III sequences; however, some of our sequences were closely related to cultivated *Deltaproteobacteria* (**Figure 3**). For example, nine sequences retrieved from the floating algal aggregate (AGG) were related (88% aa similarity) to the genus *Desulfovibrio*, a common genus in marine coastal sediments and anoxic zones (Herbert, 1999). These aggregates form in late summer below melting sea-ice (Assmy et al., 2013; Fernández-Méndez et al., 2014b) and are not connected with coastal areas at the time of formation. This could indicate that their anaerobic bacterial community does not originate from coastal sediments.

### Impact of Temperature and Nutrient Concentrations on Arctic Diazotrophs

The low temperatures and wide range of salinities present in sea ice environments shape their bacterial communities, leading to class-level differences between sea ice and water column communities (Boetius et al., 2015). To further test our first hypothesis regarding the presence of diazotrophs in the Central Arctic, we explored if the distribution and diversity of cyanobacterial and non-cyanobacterial diazotrophs was restricted by low temperatures or a specific nutrient ratio. During our cruise in August–September 2012, which covered a temperature gradient ranging from -1.7 to 3°C, nitrogen fixation genes from putative non-cyanobacterial diazotrophs were consistently amplified. This indicates that temperature limitation did not exclude non-cyanobacterial diazotrophs, an observation in agreement with previous studies (Farnelid et al., 2011; Blais et al., 2012; Díez et al., 2012). Non-cyanobacterial diazotrophs are known to thrive at low water temperatures (Riemann et al., 2010) and have been reported to be relevant for nitrogen fixation in other warmer oligotrophic oceans (Bombar et al., 2013; Moisander et al., 2014) and in the Baltic Sea (Farnelid et al., 2013). Increasing temperature seems to be linked to the structure of diazotroph community composition in the Laptev Sea samples (**Figure 4**) and could imply that a shift in surface water temperatures in the Arctic Ocean may have corresponding consequences on the regional diazotroph community. For example, non-cyanobacterial *nifH* subclusters 1A (*Geobacter*) and 2A (*Pelosinus* and *Paludibacter*) seem to be linked to increased temperatures (**Figure 4**). However, we are aware of the instability of our RDA solution due to the low number of sequences in our dataset and it is possible that deeper and more even sequencing will reveal somewhat different relationships.

The temperature ranges and optima of nitrogenase activities are only known for a few nitrogen-fixing cyanobacteria, such as *Trichodesmium* (Breitbarth et al., 2006) and *Cyanothece* (Brauer et al., 2013), which grow better at warm temperatures. Putative nitrogen-fixing cyanobacteria were only amplified in one sample in the Central Arctic Ocean (upper part of the ice at -0.2°C), supporting previous hypotheses that this group of nitrogen-fixers has not realized a niche in ice-covered polar open oceans (Murphy and Haugen, 1985; Koh et al., 2012b). However, other nitrogen-fixing cyanobacteria have been detected in snow (Harding et al., 2011; Boetius et al., 2015), glacial environments (Yallop et al., 2012; Vonnahme et al., 2015), hydrothermal vents (Mehta et al., 2003), and in other cold environments, such as Antarctic lakes (Olson et al., 1998) and sea ice (Koh et al., 2012a). Therefore, it remains unclear why they have not populated nitrogen-limited marine Arctic waters. The absence of cyanobacterial sequences in most of our samples might be due to undersampling (Supplementary Figure S4A), or to the primer bias of the primers we used toward non-cyanobacterial phylotypes (Turk-Kubo et al., 2015). However, the fact that cyanobacteria are rare in waters of the Arctic Ocean close to the ice (Lovejoy et al., 2002; Sherr et al., 2003) supports our findings.

Besides temperature, nutrient availability also plays a role in the occurrence of diazotrophs. The N:P ratios in surface waters of the Eurasian Basin range between 11 and 16 (Sakshaug et al., 2004). However, in summer the ratio can drop to 2–10, particularly in the Central Arctic north of 80°N (Supplementary Figure S2A), where nitrate availability limits algal growth (Fernández-Méndez et al., 2015). Silicate, which can also be limiting for diatom growth at the end of the productive season, showed no direct impact on *nifH* diversity. The *nifH* profile of the sample where we found cyanobacterial diazotrophs was associated with high phosphate concentrations supporting the low N:P hypothesis for autotrophic diazotrophs. In Central Arctic sea ice the occurrence of nitrogen-fixing cyanobacteria, such as *Nodularia* sp., seems to be driven by phosphate concentrations. However, as discussed previously this is a preliminary conclusion based on the very scarce data available at the moment.

We could retrieve *nifH* genes mostly of non-cyanobacterial putative diazotrophs from environments with a wide range of physical and chemical conditions, including summer sea ice with high N:P molar ratio (>16) and low salinities (0.5–3.6), and surface seawaters with low temperatures (-1.7°C) and higher salinity (33). Further research is required to determine whether any of the putative diazotrophs detected in this study are actively transcribing the *nif* operon and fixing nitrogen in the water, sea ice, and melt ponds of the Eurasian Basin.

### The Origin of Marine Diazotrophs in the Central Arctic

Our second hypothesis was that putative diazotrophs in Central Arctic sea ice would come from the coastal areas influenced by rivers, such as the Lena River. The Lena River is a source of organic matter and iron that could provide favorable conditions for nitrogen fixation in the Laptev Sea (Lara et al., 1998). Our results, however, show a distinct entire bacterial community and diazotrophic subcommunity in the Laptev Sea region, close to the Lena Delta, when compared to that present in sea ice communities (**Figure 5**).

The ice floes sampled at the end of the productive season were formed during freeze-up or in polynyas located along the fast ice edge in the Laptev and Kara seas (Supplementary Figure S5). This, together with winds that might transport dust and microorganisms offshore (Harding et al., 2011), might explain the high relative abundance of *nifH* sequences related to *Bradyrhizobium* sp. (*Alphaproteobacteria* 1K subcluster). *Bradyrhizobium* sp. is a symbiotic soil bacterium present in the nodules of leguminous plants (Hennecke, 1990), that can also fix nitrogen as free-living bacteria (Dreyfus et al., 1988), and is likely to be transported by rivers or wind. Indeed it has also been found in surface waters of the Mediterranean Sea (Le Moal et al., 2011). Some sea ice sequences clustered with the endospore-forming *Paenibacillus* (subcluster 1E) that has also been found in high Arctic soils (Jordan et al., 1978). In both environments, polar soils and sea ice, high salinities can be reached in pore fluids (Tedrow, 1966; Ewert and Deming, 2014), favoring halotolerant diazotrophs. Therefore, halotolerant diazotrophs can be expected. However, the riverine adapted freshwater diazotrophic bacteria are unlikely to thrive in the marine environment partially due to its high salinity (Fernandes et al., 1993). Subcluster 1F, which was present in both waters under the ice and those of the Laptev Sea, includes diazotrophs from the genus *Arcobacter* which is known to occur in roots of salt marsh plants, but also in sulfidic environments such as cold seeps and hydrothermal vents (Mehta et al., 2003). The dispersal of this genus from the soil of Arctic coastal areas to the Central Arctic is possible although other mechanisms might occur.

Overall, our assays of microbial communities based on the 16S rRNA gene and the *nifH* gene showed little support for the notion of the riverine origins of Central Arctic diazotrophs: the most abundant types in the bacterial and diazotrophic communities of the Laptev Sea water were notably dissimilar to those of Central Arctic sea ice and those present in the water under the ice. Sequences retrieved from brown ice (due to a high a concentration of phaeopigments from diatoms and possibly some sediments) are an exception: these clustered with sequences from the Laptev Sea waters. The subcluster represented by sequence 317LS_003 from the Laptev Sea in **Figure 3** includes sequences from sample 335BI (brown ice from station 335). Hence, a coastal (land or riverine) origin of the putative diazotrophs and other microbes in the Central Arctic is possible, but our data indicates that it is not likely the main process for diazotroph dispersal in the Eurasian Basin.

### The Distinct Diazotroph Community of the Central Arctic Ocean

To assess potential causes of dissimilarity on the basis of the limited data set available, we grouped all available sequences from the Central Arctic, coming from different environments, and compared them to sequences from other oceanic regions and polar environments such as microbial mats in Antarctic lakes or Arctic tundra soils (**Figure 6**). This comparison assumes that the variability between the different environmental types in one region has a smaller effect than the variability between regions in the phylogenetic signal of *nifH* caused by the environment type.

The Eurasian Arctic communities were more similar to the North Atlantic and Subtropical Atlantic communities than to the Canadian Arctic (**Figure 7**). This is to be expected since these oceanic regions are tightly connected through the Fram Strait. The Arctic tundra soil communities were similar to communities isolated from Antarctic lake microbial mats at the *nifH* subcluster level. These Antarctic microbial mats were described as submerged in sediments of a meltwater pond in the McMurdo ice shelf (Jungblut and Neilan, 2010), or embedded in the permanent ice cover of Lake Bonney in the Antarctic McMurdo Dry Valley in summer (Olson et al., 1998). This indicates that soil-related communities have higher similarity to one another than to marine environments with similar temperature conditions. However, the dissimilarities between the Arctic tundra, the Antarctic and the Canadian Arctic must be interpreted with caution since the *nifH* diversity in these regions is under sampled (Supplementary Figure S4B).

Despite the limitations of this preliminary comparative study due to limited data, the Central Arctic diazotrophic community appears to have a different phylogenetic composition from adjacent regions and similar cold environments (**Figure 7**). Furthermore, our *nifH* gene analysis and the ITS region analysis (**Figures 5A,B**) indicate that Central Arctic sea ice environments harbor a microbial community distinct from that of the water column. We also noted that the marine diazotrophic communities in the Arctic were distinct from the terrestrial diazotrophic communities of the Arctic tundra (Izquierdo and Nüsslein, 2006) and the Antarctic ice shelf (Olson et al., 1998; Jungblut and Neilan, 2010). This bacterial Central Arctic endemism seems to be also reflected in the eukaryotic composition after the polar night when no phototrophs are dominating (Bachy et al., 2011). The diazotrophic communities of the Eurasian Arctic north of Svalbard (Díez et al., 2012), the North Atlantic (Turk et al., 2011) and the Subtropical Atlantic (Langlois et al., 2005) appeared similar to one another. This similarity is probably due to the presence of shared phylotypes (5 CD-HIT clusters 97% nucleotide identity contained sequences from the North Atlantic and the Subtropical Atlantic) and of cyanobacterial diazotrophs in these regions (**Figure 6**). One should note that Diez et al. (2012) used slightly different primers from all other studies and this might be partially responsible for the dissimilarities between the Eurasian Basin and the other regions. As more *nifH* sequences from different Arctic environments and regions will be added to the *nifH* database, more robust conclusions can be drawn from comparisons of diazotrophic community composition among different environments. For example, although cyanobacteria have been found thriving on glaciers and snow (Harding et al., 2011; Yallop et al., 2012) no analyses of *nifH* diversity were performed during these studies. Nevertheless, our initial comparisons, which included all currently available diazotroph sequences in the Arctic, strongly suggest that the Central Arctic Ocean harbors a community of putative diazotrophic microorganisms, which is distinct to those present in other oceanic regions and similarly cold environments.

## Conclusion and Outlook

This study reveals the potential for nitrogen fixation far away from the coastal shelves in the Central Arctic, where diazotrophs were previously not detected. Nitrogen availability in the euphotic zone constrains the potential increase of primary production especially in the central oligotrophic basins (Tremblay et al., 2015). Hence it is relevant to better understand the role of nitrogen fixers. Most of the *nifH* sequences retrieved belonged to putative non-cyanobacterial diazotrophs from Clusters I and III while diazotrophic cyanobacteria were rare. The origin of the sea-ice diazotrophic community in the Central Arctic seems to be marine and not riverine, since we found significant differences between the river-influenced Laptev Sea communities, and the sea-ice related diazotrophic communities. Assuming that the upper limit of nitrogen fixation estimated by Blais et al. (2012) in the Canadian Arctic (up to 0.14 nmol N L^-1^ d^-1^ transformed to carbon using a C:N ratio of 7.3: 0.6 mg C m^-2^ d^-1^) would also take place in the Central Arctic, the nitrogen fixed by the diazotrophs could sustain ∼7% of the new primary production in the region (9.4 ± 3.6 g C m^-2^ year^-1^, Fernández-Méndez et al., 2015). However, the presence and diversity of diazotrophs does not imply high nitrogen fixation rates (Moisander et al., 2007). With current trends of warming in Arctic waters (Polyakov et al., 2010; IPCC, 2013), higher marine nitrogen fixation rates can be expected in summer ice-free waters (Blais et al., 2012). However, the real contribution of these diazotrophs to nitrogen fixation still needs to be assessed before any further conclusions can be drawn regarding their role in the ecosystem and relevance for Arctic primary production.

## Author Contributions

MF-M and AB collected the samples. MF-M, KT-K, and JR performed the laboratory molecular analysis and the phylogenetic bioinformatic analysis. MF-M, JR, and PB performed the statistical analysis. TK provided the sea ice physics background information and the ice drift analysis. AB and JZ designed the study, organized the analysis and contributed to the discussion of the results. MF-M prepared the manuscript with contributions from all-coauthors.

## Conflict of Interest Statement

The authors declare that the research was conducted in the absence of any commercial or financial relationships that could be construed as a potential conflict of interest.

## Footnotes

1 http://www.ecology-research.com
