# Supplementary material for: Diazotroph Diversity in the Sea Ice, Melt Ponds, and Surface Waters of the Eurasian Basin of the Central Arctic Ocean
Source: Front Microbiol. 2016 Nov 23;7:1884. doi: 10.3389/fmicb.2016.01884 (PMC5120112; doi:10.3389/fmicb.2016.01884)

Supplementary Material

Diazotroph diversity in the sea ice, melt ponds and water column of the Central Arctic Ocean

Fernández-Méndez et al. Submitted to Frontiers Aquatic Microbiology in June 2016

Table S1. Samples collected and analyzed for *nifH* gene presence.

Station	Latitude	Longitude	Date	Environment	Volume filtered (ml)	Number of *nifH* sequences retrieved
218	82°59.400'N	30°3.230'E	07.08.2012	Surface water (CTD)	2000	3
224	84°3.030'N	31°6.830'E	10.08.2012	Melt Pond 3 Water	1000	11
224	84°3.030'N	31°6.830'E	09.08.2012	Water under the ice	2000	0
224	84°3.030'N	31°6.830'E	10.08.2012	Melt Pond 1 Aggregates	100	0
224	84°3.030'N	31°6.830'E	10.08.2012	Melt Pond 2 Water	1000	0
224	84°3.030'N	31°6.830'E	10.08.2012	Melt Pond 2 Aggregate	50	0
224	84°3.030'N	31°6.830'E	10.08.2012	Open water aggregates	30	43
224	84°3.030'N	31°6.830'E	09.08.2012	Ice Top	2000	39
224	84°3.030'N	31°6.830'E	09.08.2012	Ice bottom	2000	6
234	83°59.400'N	39°28.420'E	12.08.2012	Surface water (CTD)	2000	0
237	83°59.190'N	78°6.200'E	15.08.2012	Melt Pond 1	2000	0
237	83°59.190'N	78°6.200'E	16.08.2012	Ice Top	2000	38
237	83°59.190'N	78°6.200'E	16.08.2012	Ice bottom	2000	0
245	83°55.140'N	75°58.890'E	19.08.2012	Surface water (CTD) 20 m	2000	16
255	82°40.240'N	109°35.370'E	21.08.2012	Melt Pond 3 Water + yellow/white aggregates	2000	0
255	82°40.240'N	109°35.370'E	22.08.2012	Ice Top	1800	7
255	82°40.240'N	109°35.370'E	21.08.2012	Water under the ice	800	0
255	82°40.240'N	109°35.370'E	22.08.2012	Ice bottom	2000	0
263	83°4.730'N	110°8.990'E	22.08.2012	Surface water (CTD)	1500	3
277	82°52.950'N	130°7.770'E	27.08.2012	Water under the ice	1000	0
277	82°52.950'N	130°7.770'E	27.08.2012	Ice top	1700	33
277	82°52.950'N	130°7.770'E	27.08.2012	Ice bottom	2000	6
277	82°52.950'N	130°7.770'E	27.08.2012	Melt Pond 3 water + 500 ml white aggregates	1000	0
308	77°10.290'N	114°55.200'E	01.09.2012	Surface water Laptev Sea (CTD)	2000	10
311	77°23.810'N	118°11.750'E	01.09.2012	Surface water Laptev Sea (CTD)	2000	1
314	77°42.950'N	118°18.990'E	01.09.2012	Surface water Laptev Sea(CTD)	2000	18
316	78°21.000'N	118°36.000'E	02.09.2012	Surface water Laptev Sea (CTD)	2000	7

317	78°39.980'N	118°44.580'E	02.09.2012	Surface water Laptev Sea (CTD)	2000	4
319	79°9.750'N	119°47.080'E	02.09.2012	Surface water Laptev Sea (CTD)	2000	0
323	81°55.530'N	131°7.720'E	04.09.2012	Water under the ice	2000	1
323	81°55.530'N	131°7.720'E	04.09.2012	Ice Bottom	2000	0
323	81°55.530'N	131°7.720'E	04.09.2012	Melt Pond 1 Water	2000	0
323	81°55.530'N	131°7.720'E	04.09.2012	Ice Top	1500	4
329	81°52.550'N	130°52.650'E	05.09.2012	Surface water (CTD)	2500	0
335	85°6.110'N	122°14.720'E	12.09.2012	Melt Pond 4	2000	0
335	85°6.110'N	122°14.720'E	09.09.2012	Melt Pond 1 water	2000	0
335	85°6.110'N	122°14.720'E	09.09.2012	Ice Top	1800	3
335	85°6.110'N	122°14.720'E	09.09.2012	Ice Bottom	2000	38
335	85°6.110'N	122°14.720'E	16.09.2012	Brown Ice due to high algal biomass	2000	41
341	85°9.540'N	123°21.540'E	09.09.2012	Surface water (CTD)	2000	42
349	87°56.010'N	61°13.040'E	20.09.2012	Ice Top	2000	46
349	87°56.010'N	61°13.040'E	20.09.2012	Ice Bottom	2000	0
349	87°56.010'N	61°13.040'E	20.09.2012	Brown Ice due to high algal biomass	250	0
349	87°56.010'N	61°13.040'E	21.09.2012	Melt Pond 3 water	2000	4
349	87°56.010'N	61°13.040'E	21.09.2012	Water under the ice	2000	0
360	88°49.660'N	58°51.810'E	23.09.2012	Water under the ice	2000	6
360	88°49.660'N	58°51.810'E	23.09.2012	Melt Pond 3 water	2000	9
360	88°49.660'N	58°51.810'E	23.09.2012	Ice Top	2000	41
360	88°49.660'N	58°51.810'E	23.09.2012	Ice Bottom	2000	0
360	88°49.660'N	58°51.810'E	25.09.2012	Brown Ice due to high algal biomass	2000	0
377	87°12.640'N	51°50.580'E	25.09.2012	Surface water (CTD)	2000	49
384	84°22.490'N	17°27.220'E	30.09.2012	Ice	2000	0
384	84°22.490'N	17°27.220'E	30.09.2012	New Ice	800	0

Table S2. Unifrac unweighted distances.

***Distances between relatively well represented regions according to the rarefaction curves in Figure S4B.**

Unifrac Distance	Central Arctic	Canadian Arctic	Eurasian Arctic	Arctic Tundra	Antarctic	North Atlantic
Canadian Arctic	0.7617					
Eurasian Arctic	0.8315	0.6392				
Arctic Tundra	0.8239	0.5831	0.6730			
Antarctic	0.8021	0.6255	0.6454	0.5144		
North Atlantic	0.7869*	0.7141	0.6885	0.7275	0.6779	
Subtropical Atlantic	0.8544*	0.6896	0.6100	0.7642	0.7586	0.6783

Figure S1: Surface water temperature during ARKXXVII/3 cruise in August-September 2012. Temperature measured in the water column at 5 m depth. Stations with temperatures below -1°C were ice covered, while stations with temperatures above 0 °C correspond to open waters. Data available in the database PANGAEA (Rabe et al., 2012). Bathymetry of the Arctic Ocean from Ocean Data View (Schlitzer, R., 2015).

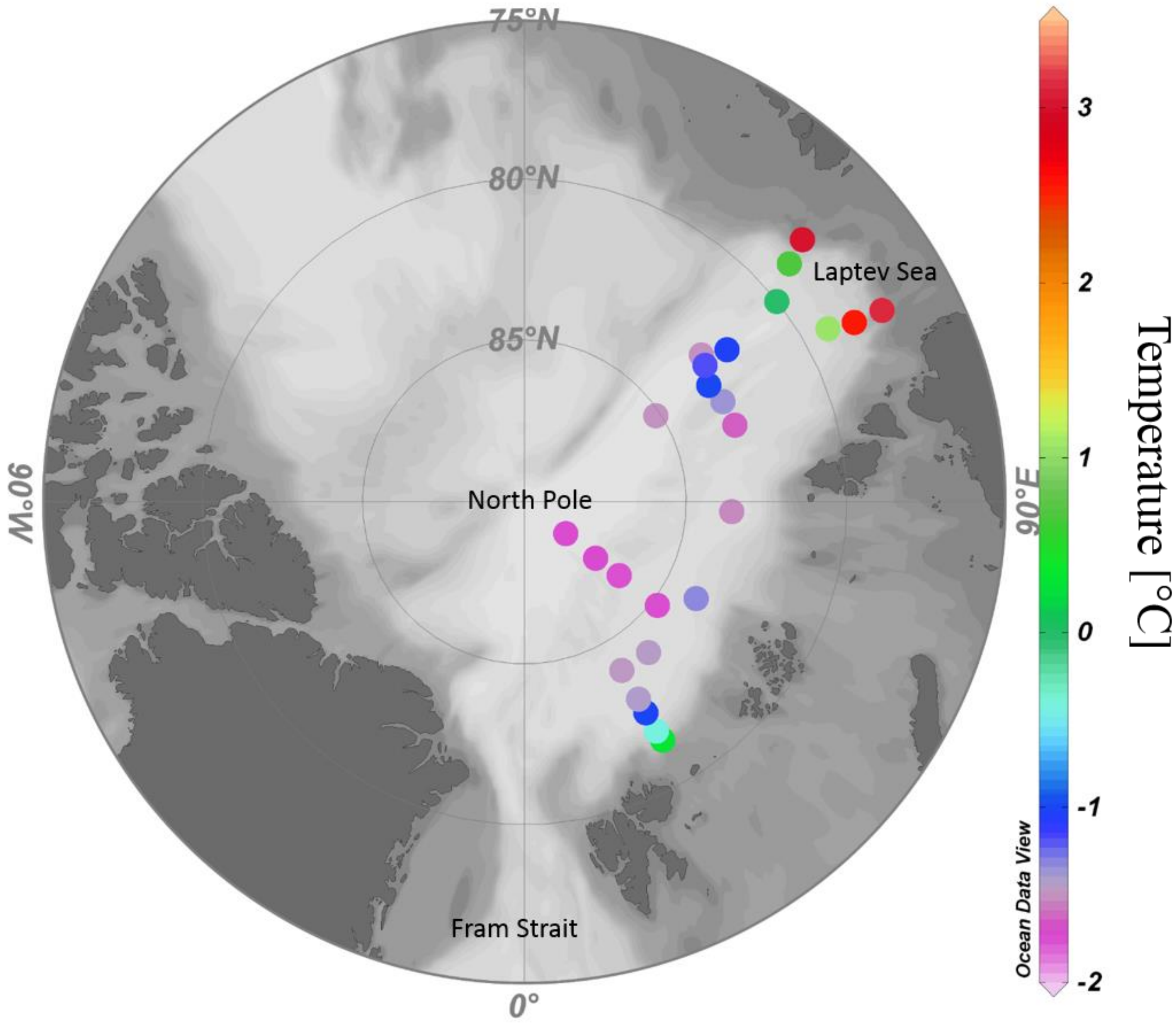

Figure S2: Nitrogen to phosphorous molar ratios in the water column (A) and sea ice (B). Values were calculated for the integrated euphotic zone (1% incoming irradiance) in the water column and for the average sea-ice thickness at each ice station. Two different nutrient regimes were identified for the water column: N:P <5 in the Amundsen Basin close to the North Pole; and N:P ~10 in the Nansen Basin along the ice edge and in the Laptev Sea open waters. The data can be found in PANGAEA (Fernández-Méndez et al., 2014). Bathymetry of the Arctic Ocean from Ocean Data View.

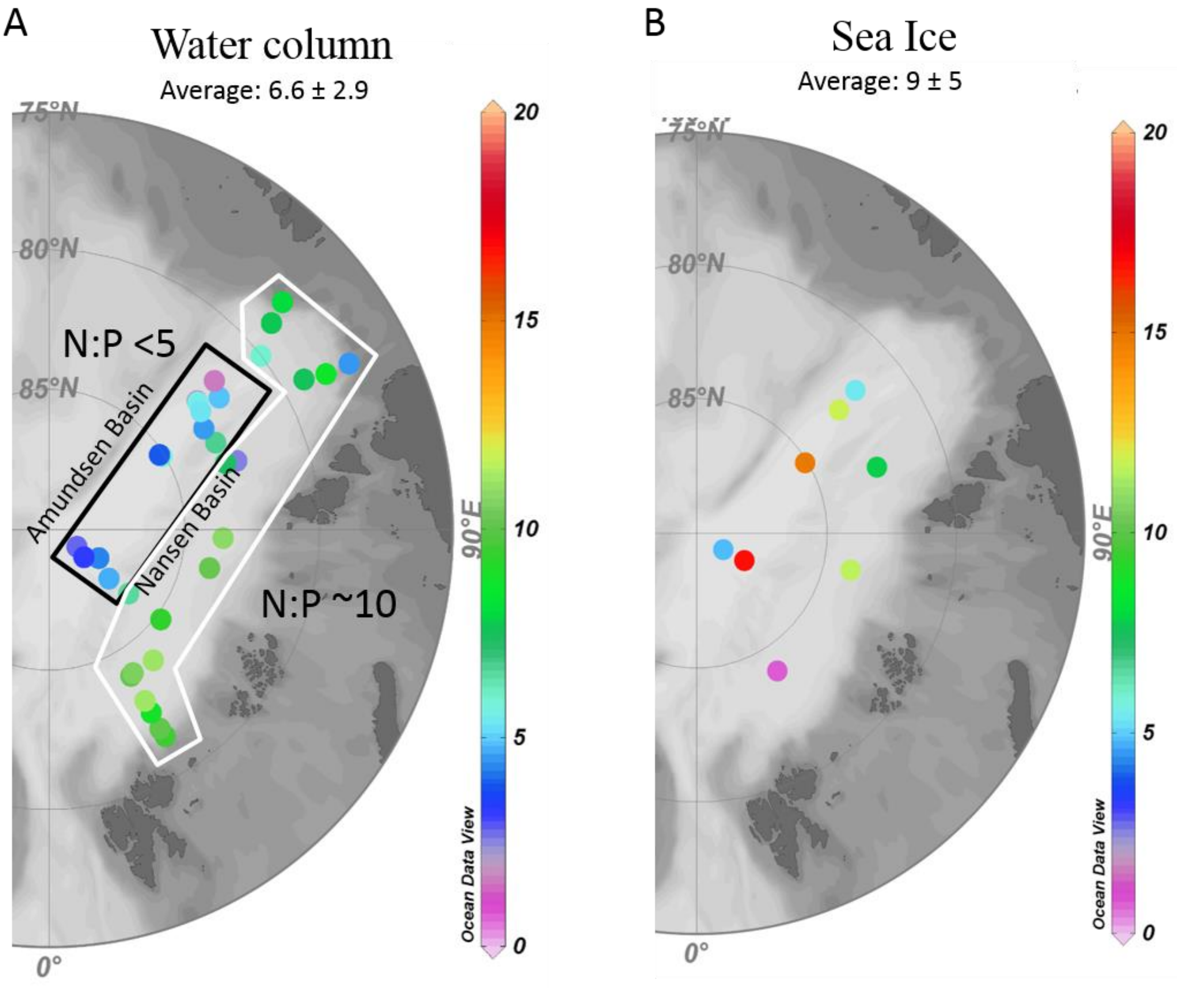

Figure S3: Histogram of residuals of the RDA analysis shown in Figure S1.

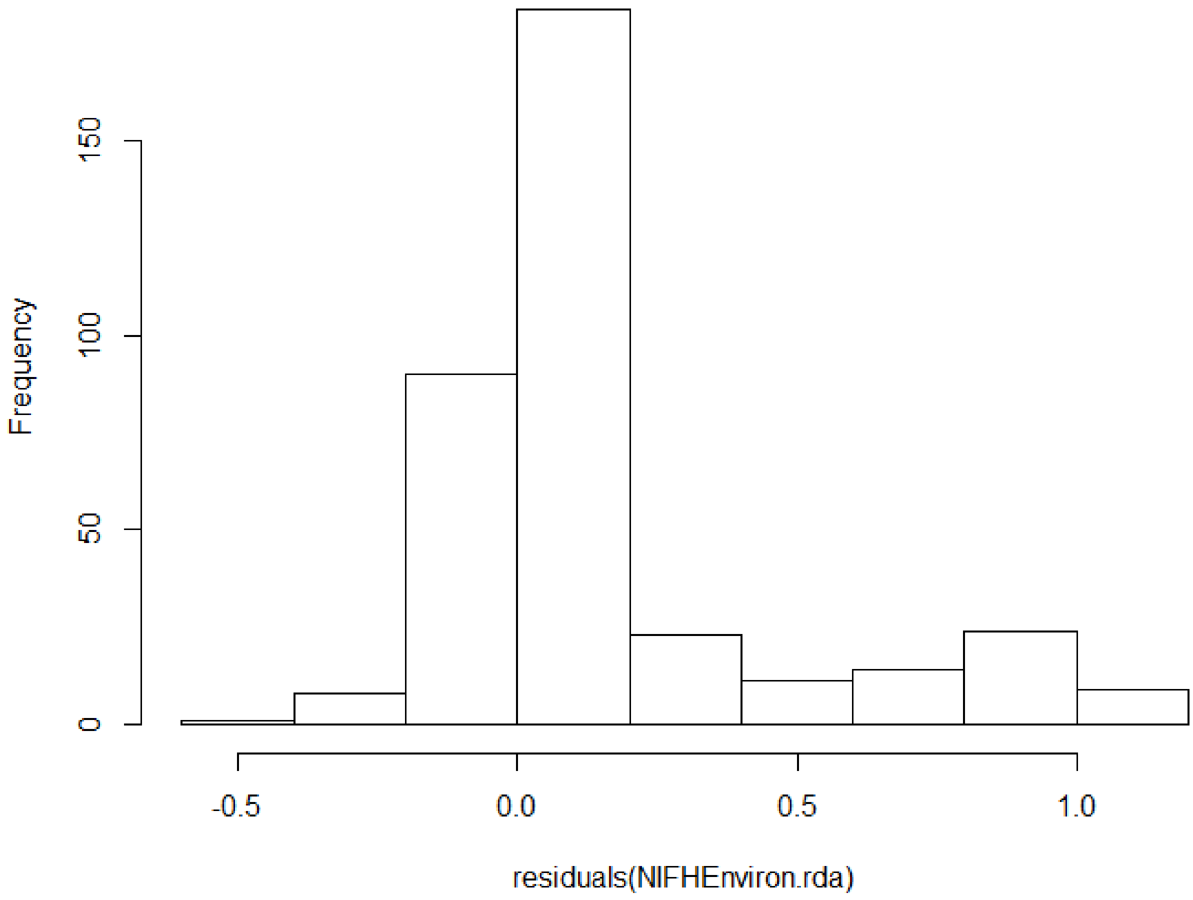

Figure S4: Rarefaction and extrapolation with Hill numbers

(A) Rarefaction curves of the 92% aminoacid similarity representative sequences of the different Central Arctic environments sampled.

The numbers on top of the three panels represent the Hill numbers. Hill number 0 refers to species richness, 1 to the number of 'typical' species in the community (exponential of Shannon entropy), and 2 to the number of very abundant species in a community (inverse Simpson).The solid lines represent interpolation and the dashed lines extrapolation. The shaded area is the 95% confidence interval calculated with 50 repetitions using the iNEXT package in R.

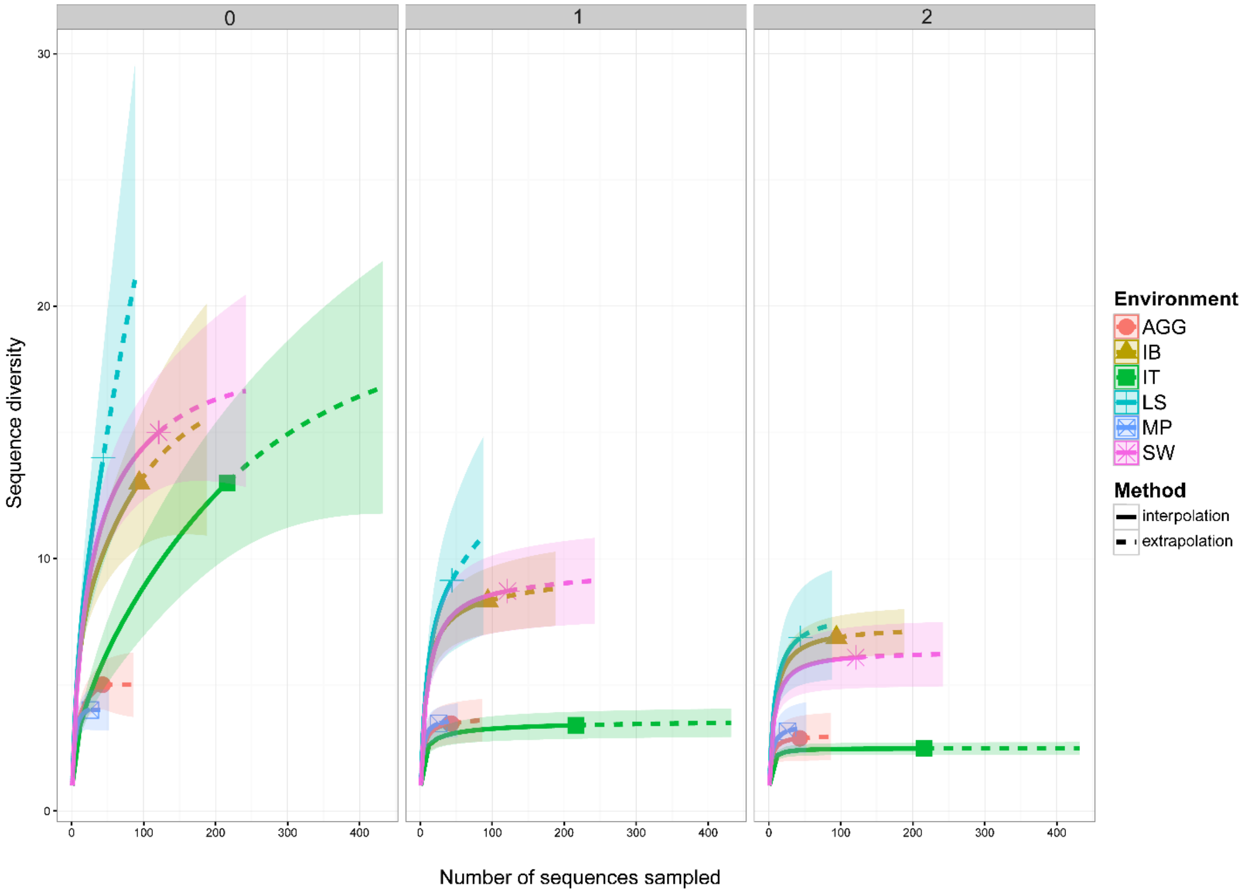

(B) Rarefaction curve of 97% nucleotide identity representative sequences of the different oceanic regions.

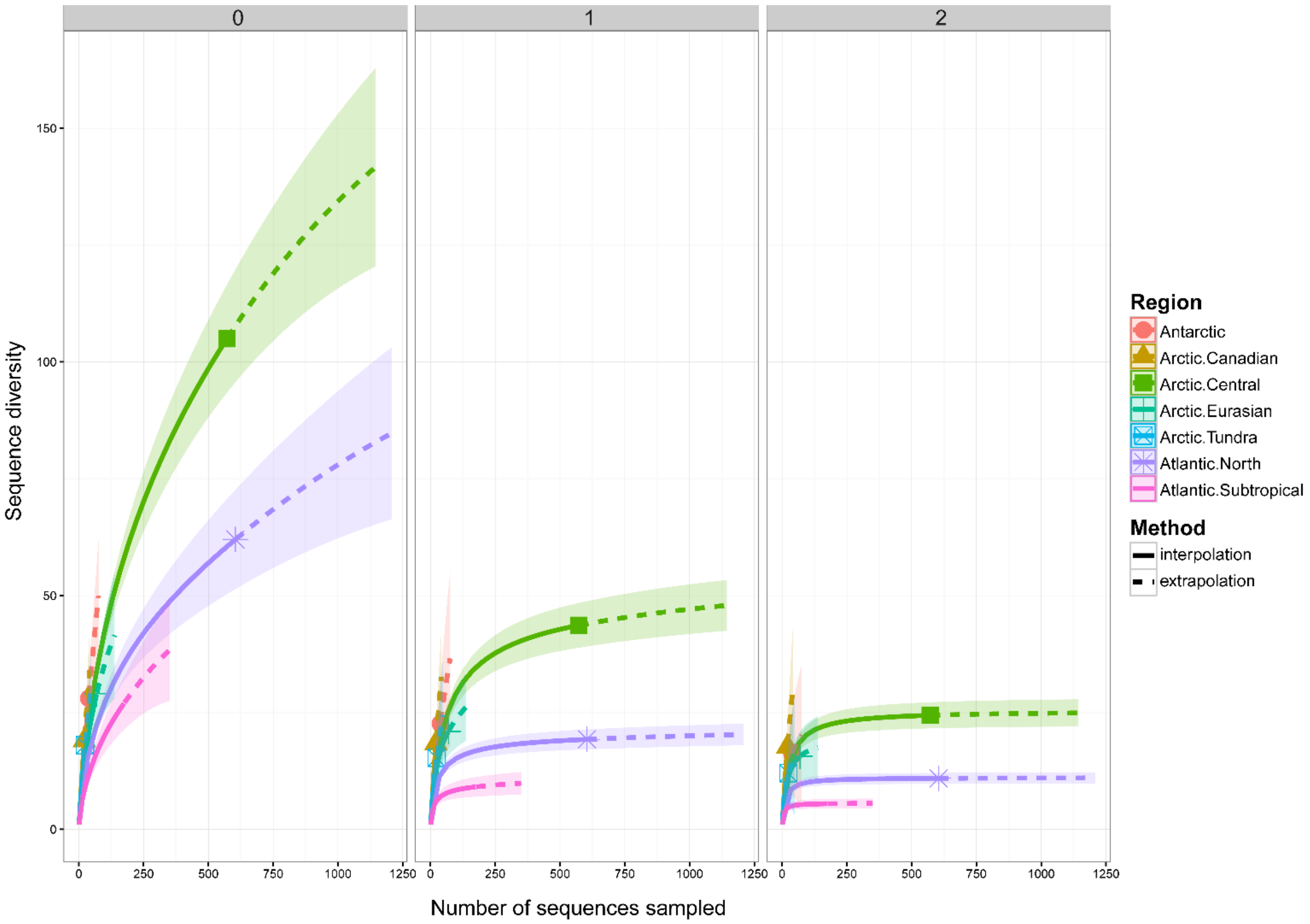

Figure S5: Ice flow tracking of ice floes sampled in summer 2012.

Grey lines represent the track of the ice floes sampled in summer 2012 from their formation close to the coast to the moment of sampling.

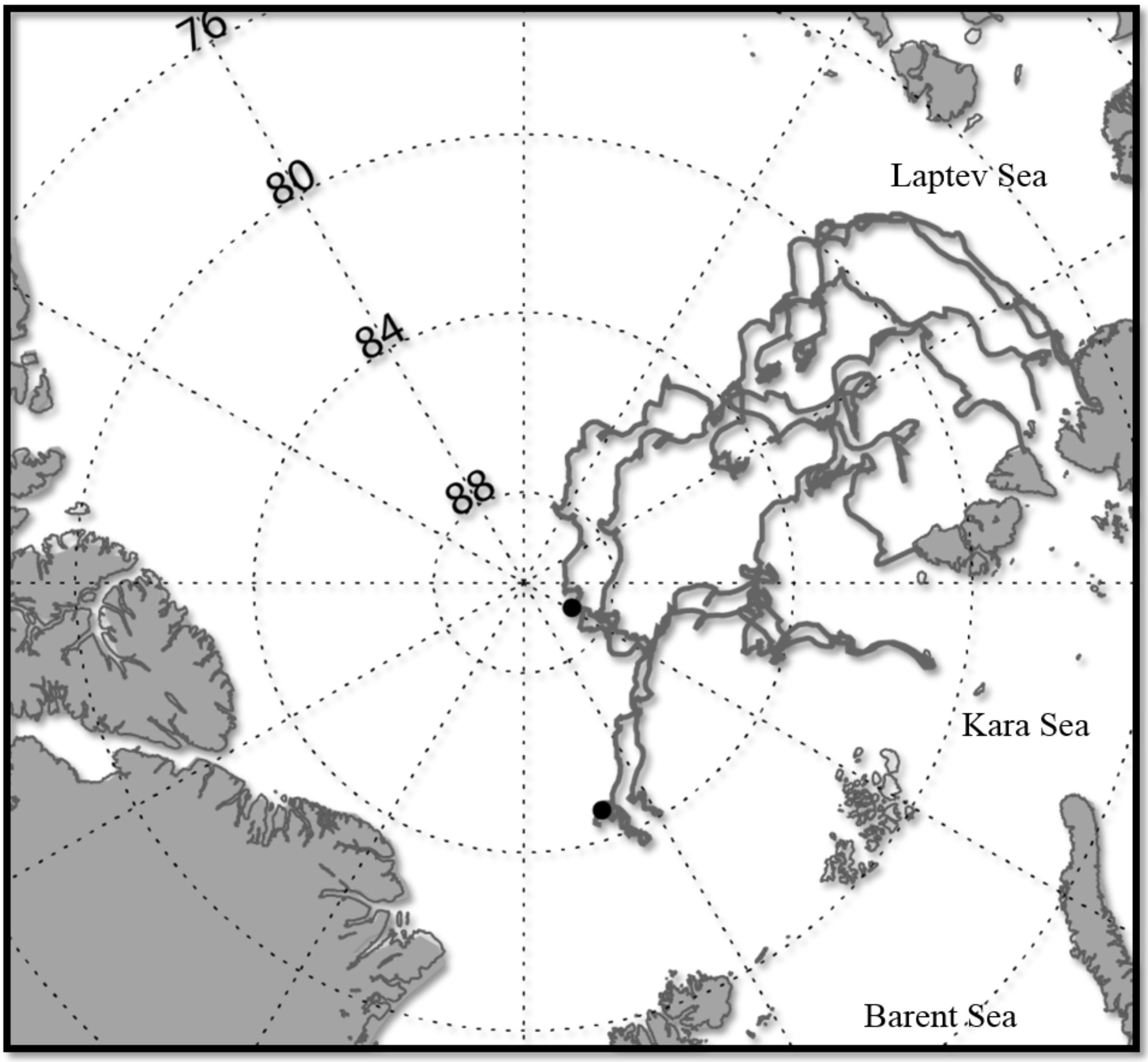

Supplement: Supplementary file 1 [file Data_Sheet_1.pdf]
